# Using mobile eye tracking to study dogs’ understanding of human referential communication

**DOI:** 10.1098/rspb.2024.2765

**Published:** 2025-02-12

**Authors:** Christoph J. Völter, Karoline Gerwisch, Paula Berg, Zsófia Virányi, Ludwig Huber

**Affiliations:** ^1^ Comparative Cognition, Messerli Research Institute, University of Veterinary Medicine Vienna, Medical University of Vienna and University of Vienna, Vienna, Austria; ^2^ Department of Comparative Cultural Psychology, Max Planck Institute for Evolutionary Anthropology, Leipzig, Germany

**Keywords:** canine cognition, human–animal interaction, pointing, gazing, mobile eye tracking, referential communication

## Abstract

The extent to which dogs understand human referential communication is among the most studied questions in canine cognition research. While it is widely accepted that dogs follow (some) human referential signals, the way they understand them remains controversial. Here, we applied mobile eye tracking with dogs to investigate during real-world interactions how ostensive pointing and gaze cues direct dogs’ visual attention and bias their subsequent choices in an object-choice task. We addressed the question of whether dogs would exhibit a greater response to referential communication compared with other directional cues. Five conditions were tested (pointing, pointing + gazing, gazing, fake throwing and no-cue control), each cue condition indicating the location of a hidden food reward. Results demonstrated that the combination of pointing and gazing significantly increased dogs’ attention towards the designated referent. In pointing + gazing, dogs maintained longer attention on the referent compared with other conditions and they approached it significantly above chance levels. While the alternative cue (fake throwing) moved the dogs’ gaze to the indicated direction, it did not increase the frequency of gaze shifts to the precise referent location. Our findings highlight that the joint use of pointing and gazing is a particularly effective method for directing dogs’ attention to a referent.

## 1. Introduction

Referential communication, an early developing aspect of human interaction, plays a central role for us humans when coordinating with other agents [1]. It enables the communicator to direct the attention of their counterpart towards external referents. According to reviews of the pointing literature [2,3], among non-human animals, dogs (*Canis familiaris*) show a particularly robust inclination to follow at least some forms of human referential communication. However, a number of studies suggest that the rich experience of many dogs with humans might explain their success and not primarily domestication. This is evidenced by the relatively poor performance of dogs with less exposure to humans such as shelter dogs [4–9].

In human communication, ostensive signals that inform the recipient about the communicative nature of the subsequent acts play an important role from an early point in ontogeny [10]. Sensitivity to ostensive signals allows individuals to recognize when an agent intends to convey a message [11,12]. Dogs, too, seem to be sensitive to ostensive signals such as eye contact and verbal addressing (e.g. [13–23]). They follow directional gaze cues and gestures with their gaze as well as with their subsequent choice behaviour more frequently after they have been addressed ostensively [16,22,24], and ostensive attention calling can even lead them to less efficient or erroneous choices similar to what has been found with human infants [13,15,25]. For instance, Téglás *et al*. [22] found in an eye-tracking study that ostensive addressing, rather than an arbitrary attention-getter highlighting the signaller’s head, triggered higher rates of gaze following in dogs.

It has been suggested based on these findings that dogs respond to human directional communication selectively, either reflexively or by understanding human communicative intentions to some extent [13,20]. This does not necessarily mean, however, that dogs comprehend human directional signals as referential communication. Alternatively, it has been proposed that dogs may interpret the pointing gesture as an imperative directive that sends them in a certain direction [26]. To put it differently, the finding that ostension matters for dogs’ comprehension of human communication, leaves open the question *what* dogs encode: target location or target identity. To address this question, after calling the dogs’ attention and pointing at one out of two toys, Tauzin *et al*. [27] swapped the object locations. As the dogs exhibited a preference for the cued location over the cued object, they suggested that dogs primarily encode the target location rather than its identity. Additionally, the finding that dogs sometimes follow referential cues even when they know that the food reward has been hidden elsewhere and that the cued hiding location is empty ([15,28,29] but see [30]) seems to support the imperative interpretation hypothesis. Other findings, however, suggest that dogs expect that there is a referent when seeing a pointing gesture. When Scheider *et al*. presented dogs with pointing gestures to an empty location, dogs reacted with a sustained exploration of the cued area suggesting that they expected to find something there [31]. Also, Soproni *et al*. [21] found that dogs after some pointing training (like human children but unlike chimpanzees [32]) followed human gaze cues that were directed at a target bowl but not when the gaze cue was directed above the target bowl, suggesting that directionality in itself is not sufficient to cue dogs to a specific target. Following this approach, in the current study, we set out to investigate whether dogs follow humans’ referential communication more than their other directional cues.

Previous gaze and point following studies either focused on dogs’ subsequent choice behaviour [33] or coded shifts in the dogs’ visual attention based on visible head turns [17] or based on manual scoring of the gaze direction [34] (with the exception of the aforementioned eye-tracking study by Téglas *et al*. [22] that might, however, lack external validity due to the screen-based nature of the stimuli). Measuring head turns or gaze shifts via regular video recordings and manual scoring is not a very precise method for determining the exact gaze location. Mobile eye tracking [35–37] can be used to acquire detailed, precise and valid data on dogs’ visual attention in response to human referential communication. By tracking their eye movements, we can observe how quickly, reliably and accurately dogs follow human referential signals providing insights into the cognitive processes involved in interpreting and responding to referential communication.

In the current study, we therefore assessed pet dogs’ (*n* = 20) responsiveness to different types of referential communication by means of mobile eye tracking. Specifically, we investigated dogs’ immediate gaze shifts following directional cues as well as their subsequent choice behaviour. We addressed the question of whether dogs would follow referential communication more than another directional action with their gaze on the indicated referent (and not just roughly in the indicated direction). In an object-choice task, we presented them with directional cues that indicated where a food reward was hidden, while they wore the mobile eye tracker. We presented the dogs with five conditions (pointing, pointing + gazing, gazing, fake throwing and no-cue control), four of which provided the dogs with a directional cue indicating the location of the hidden food reward. One condition, the throwing condition, involved a directional action that is not usually considered as referential communication. We predicted higher gaze shifting rates following pointing + gazing than throwing. We also predicted that pointing would be more effective than gazing [16] and that especially the combination of gazing and pointing signals would elicit a shift of the dogs’ gaze to the signalled referent.

## 2. Material and methods

### (a) Subjects

We tested 20 pet dogs (8 mixed breeds, 3 American Staffordshire Terriers, 2 Australian Shepherds, 2 Poodles, 1 Collie, 1 Flat Coated Retriever, 1 German Shepherd, 1 Rhodesian Ridgeback, 1 Parson Russel Terrier; mean age: 56.0 months, range: 13−153 months; 9 females, 11 males). We selected only dogs with a head (35–44 cm) and muzzle (22–29 cm) circumference appropriate for the size of the mobile eye-tracking headgear. Nine additional dogs started but did not complete the training (three dogs did not accept the goggles, three owners did not have time to complete the training, one dog dropped out due to poor health and two dogs were too aroused when they were in the testing rooms).

### (b) Eye tracking

We used the DB9-K9-Canine Headgear mobile eye-tracking system (Positive Science, USA) to record the dogs’ eye movements. The headgear was connected to the recording unit, which was attached to the dog’s harness during recordings. The headgear consisted of Rex Specs V2 goggles (size: large) with a microphone and two cameras attached to it. One camera, the eye camera, was mounted on a wire arm connected to the right rim of the goggles, aimed at the dog’s right eye. The other camera, the scene camera, was mounted on the upper rim of the goggles capturing the scene in front of the dogs.

Next to the eye camera, an infrared emitting diode (IRED) produced the corneal reflection, which helped account for subtle headgear movements. We adjusted the position of the eye camera and IRED for each subject to ensure that the right eye was in view and properly illuminated. The scene camera recorded a field of view measuring 101.55° horizontally and 73.60° vertically. The two cameras recorded videos at a frame rate of 29.96 frames per second.

### (c) Habituation

In preparation for the study, the caregivers habituated their dogs to the headgear at home over the course of short sessions spread over multiple weeks. The caregivers conducted the habituation in a slow, stepwise manner exclusively using positive reinforcement. Initially, the dogs became accustomed to wearing the goggles without any add-ons. Once they were comfortable with that, we replaced the goggles with a dummy version of the eye tracker, including camera, cable and recording unit replicas. Dogs participated in the tests only after successfully completing this multi-week habituation process (for more details on the training procedure, see the electronic supplementary material).

### (d) Design

In a within-subject design, we presented the dogs with five conditions (gazing, pointing, pointing and gazing (henceforth: *p* + *g*), fake throwing, control; see electronic supplementary material, video S1) with six trials per condition. Overall, we conducted 30 trials, split into two sessions of 15 trials each conducted on different days. We presented the conditions in blocks except for the control condition, which was interspersed within the other conditions and took place in both sessions (three control trials per session). We did not present the control condition in a blocked way to avoid frustration induced by multiple trials in a row in which there was no obvious cue available concerning the location of the food reward. We counterbalanced the order of test conditions across individuals. After each block, there was a short break. We pseudorandomized the location of the food reward across trials with the restriction that the same bowl was not baited more than twice in a row. Throughout the test, we baited only one of the two bowls and the directional cues were always directed towards the baited side.

### (e) Set up and procedure

The study took place at the Clever Dog Lab, University of Veterinary Medicine Vienna. In each session, the handler (H; usually the dogs’ caregiver) sat on a chair facing the experimenter (two female experimenters collected the data). The dog sat or stood in front of the owner in a marked area (55 × 40 cm, distance to the experimenter: 170 cm; henceforth: starting position). The experimenter kneeled on a kneepad. In front of the experimenter (E), we added a 140 cm long tape mark to the floor (at a distance of 20 cm from E and 150 cm from the dogs’ starting position) to standardize the positions of the bowls in the test phase. We used four cameras mounted to the walls of the room to record the sessions.

#### (i) Warm-up and calibration

Each session started with a warm-up phase (without the eye-tracking headgear), in which the dogs could freely explore the test room for 3 min. Next, the headgear was put on the dog and the camera position was adjusted. Then we conducted a first calibration procedure, followed by the familiarization, the test phase and a second calibration procedure at the end of the session. If the headgear or the eye camera moved in any obvious way during the test, we conducted further calibrations throughout the session.

For the eye-tracking calibration procedure, we placed a wooden panel (100 × 100 cm) on the tape mark, occluding the experimenter from the dogs’ perspective. The panel contained five holes (diameter: 5 cm) that were covered by black felt patches on the experimenter’s side (to reduce the likelihood that the dogs would look at the holes even when they were not intentionally cued by the experimenter). The purpose of the calibration procedure was to identify the dogs’ gaze location during the subsequent (offline) calibration that was part of the post-processing. During the calibration procedure, the dogs sat centrally in front of H facing the calibration panel and E crouched behind it. E then lifted a felt patch covering one of the holes, addressed the dogs by their names and showed a dog toy by poking it through the hole. Additionally, E looked out on all three sides of the wall while calling the dog’s name. E also verbally confirmed when she could see the dogs looking at her; this verbal signal was used as an auxiliary cue during the offline calibration.

#### (ii) Familiarization

After the calibration procedure, E familiarized the dogs with two opaque bowls covered by paper lids (one dark green and one bright green) in which we hid the food rewards during the test phase (both bowls were rubbed with sausage at the start of the session). In the familiarization, we only presented the dog with one bowl at a time. H held the dog at the starting line. E kneeled approximately 20 cm behind the tape mark on top of which she placed the bowl. E made eye contact with the dog, called the dog by its name and then baited one of the bowls while the dog was watching. E retrieved the food reward from behind her back, held it up centrally with both hands to show it to the dogs, placed it in the bowl and covered it by means of the paper lid. The two bowls and locations were baited equally often during the familiarization phase. E slit the bowl to its final position (at one of the ends of the tape mark). On a signal by E, H released the dog. E praised the dog for retrieving the treats throughout the familiarization phase. We conducted four familiarization trials before moving on to the test phase.

#### (iii) Test phase

**Figure 1.**
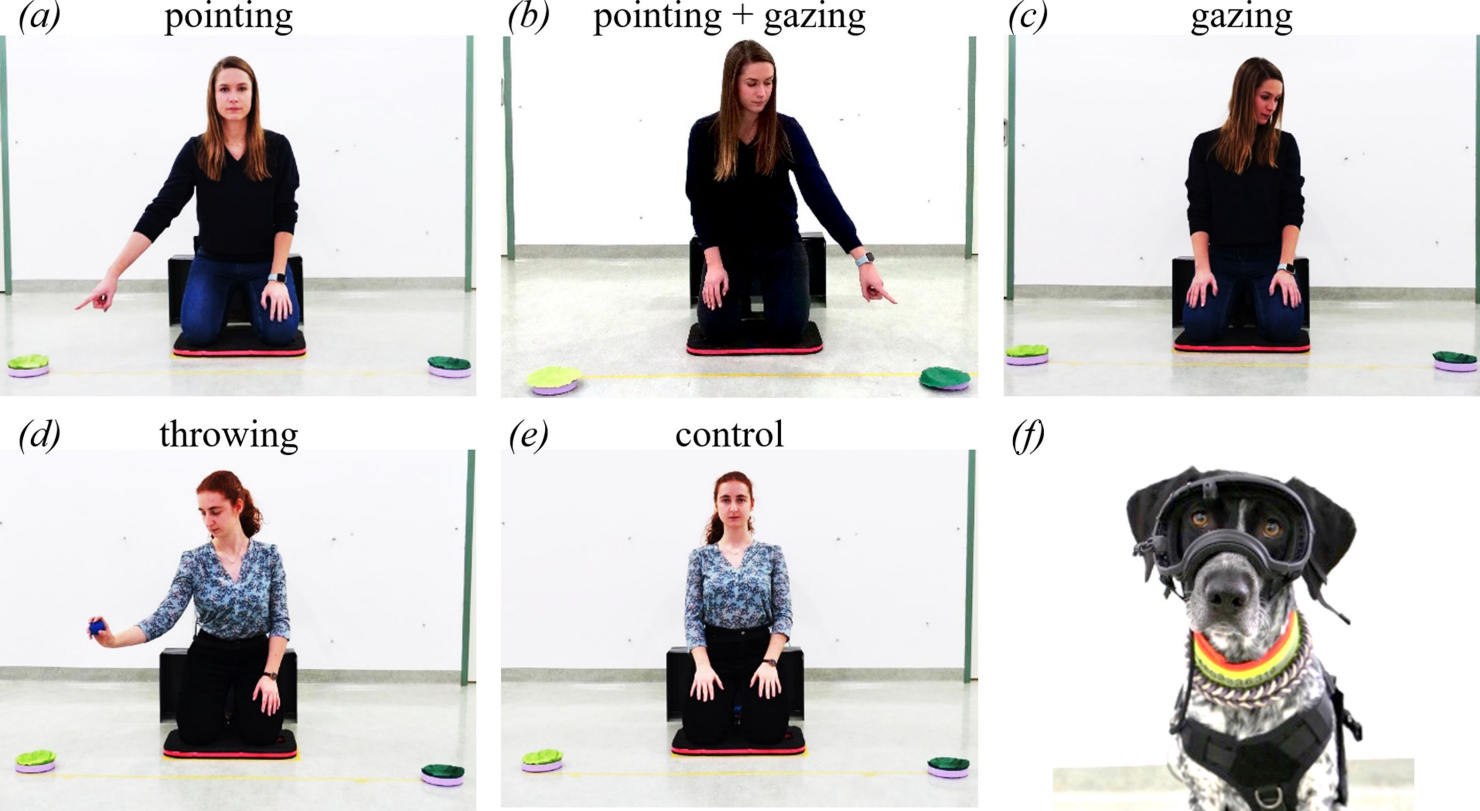
The cues provided in the test trials (signalling phase): (*a*) pointing, (*b*) pointing + gazing, (*c*) gazing, (*d*) (fake) throwing, (*e*) control condition. (*f*) A dog wearing the eye-tracking headgear.

In the test trials, E placed both bowls centrally in front of her, one in front of the other. Then she placed an occluder (h × w × d: 30 × 59 × 21 cm) in between the dog and the bowls to occlude the baiting. She now baited one of the bowls behind the occluder in the same way as in the familiarization phase. Then she removed the occluder, put it behind her back and slid the two bowls simultaneously to their end positions at the ends of the tape mark (140 cm apart). In all conditions, the experimenter then addressed the dog by looking at the dog and calling the dog’s name (addressing phase). Thereafter, the signalling phase started that varied depending on the condition (figure 1). In the control condition, the experimenter just maintained her pose (looking straight at the dog) whereas in the test conditions she gave a directional cue toward the baited side.

In the pointing condition, E pointed at the target bucket (using an ipsilateral, momentary, proximal pointing gesture, the distance between the pointing hand and bowl *ca* 15−30 cm). The pointing gesture was a direct movement of the ipsilateral arm (left arm to left bowl and *vice versa*) from the resting position on the side of the thigh to the baited bowl. E’s torso remained facing squarely forward during the pointing gesture. E held the pointing position for 3 s and then returned to her resting position with the hands on the side of the thigh. In the gazing condition, E looked from the dog to the baited bowl (3 s), back to the dog, again to the baited bowl (3 s) and finally looked back to the dog. In the pointing + gazing (*p* + *g*) condition, E pointed to the referent while looking at it (the pointing gesture was identical to the pointing condition). In the (fake) throwing condition’s addressing phase, E looked at the dog and then retrieved a toy ball from a pocket behind her back, and moved the ball in the line of sight between E and the dog. In the subsequent signalling phase, E pretended to throw it towards the baited bowl and then within the same movement put it back behind her back (one experimenter briefly looked at the target object, a second experimenter did not; however, the experimenter ID did not significantly affect the dogs’ gaze shifts to the correct bowl; see electronic supplementary material for an analysis of the potential impact of experimenter ID on the dogs’ performance). In all four cueing conditions, the directional signal was a reliable indicator of the location of the hidden food.

After the signalling, E said ‘now’ in a low monotone voice to signal H to release the dog and to say ‘okay’ (or another release command familiar to the dog). Until this point, H held the leash while wearing a blindfold such that H was ignorant of the location of the reward. The dogs could now approach one of the bowls and remove the lid. Once the dogs made contact with one of the bowls E removed the other bowl to prevent the dogs from making a second choice. If the dogs chose the baited bowl first, they were allowed to eat the reward.

### (f) Scoring and analysis

We scored whether the dogs first approached the correct or incorrect bowl within 20 s of release. Trials in which the dogs did not approach a bowl within 20 s were scored as ‘no choice’ and excluded from the analysis (see below).

We employed the software Yarbus (PositiveScience) for synchronizing the eye and the scene camera recordings, detecting the two landmarks (pupil and corneal reflection) required for the estimation of the gaze location, and conducting the offline calibration and validation. In the offline calibration, we selectively chose time points within the recordings for which we had a high degree of confidence regarding the dog’s gaze location (e.g. using E’s verbal confirmation when she could see the dog looking at her).

We exported from Yarbus the synchronized recordings with the overlaid gaze location and a data file with the gaze coordinates. Frames in which either the pupil or corneal reflection was not detected were excluded from further analysis (the landmarks were detected, on average, in 88.8% of the frames, range by recordings: 70.4–98.5%). We segmented the gaze coordinates in fixations (minimum fixation duration: 100 ms) and categorized the fixations into areas of interest using the program GazeTag (PositiveScience). We categorized only fixations for which the pupil and the corneal reflection were detected accurately. We categorized the interest areas based on the object marked by the gaze overlay onto the experimenter’s face, hand, body, correct and incorrect bowl. If the gaze location did not cover an object of interest (e.g. when it was in the white wall in the background) we used the fixation thumbnail (a 128 × 128 pixel area determined at the middle of every fixation) and assigned it to the closest object of interest visible in that area.

We also segmented the synchronized recordings into interest periods using the Loopy scoring tool (Loopbio, AT). We focused our analysis on the addressing and signalling phases. The addressing phase began when E addressed the dogs by saying their name while making eye contact. The addressing phase ended with the start of the signalling phase. Depending on the condition, the signalling phase began when E either moved her head or hand in the indicated direction. In the control condition, the signalling phase began after the verbal addressing ended. The signalling phase ended when E gave the command to H to release the dog.

Ten trials were excluded from the analyses (1.7% of all trials) for various reasons, including three trials repeated by mistake, two trials in which the dog was released prematurely by H, two trials in which the dog did not make a choice within 20 s after the release and three trials that were not recorded by the eye tracker. In 19 additional trials (3.2% of all trials) the addressing phase was not clearly distinguishable from the signalling phase and was therefore omitted from the addressing phase analysis. One dog by mistake did not receive the gazing condition and 12 (instead of 6) *p* + *g* trials instead.

We merged the GazeTag data including the tagged fixations (areas of interest) with the Loopy data, which contained the interest periods, and aggregated the data by calculating the sum of fixations within each interest period and area of interest. Next, we added zero values representing trials in which the dogs were not looking into any area of interest.

As part of the validation, we selected time points from the calibration procedure that were not subsequently used for offline calibration. We selected between 6 and 14 points for the validation per recording and calculated the average deviation between manually determined coordinates and reconstructed coordinates based on the calibration. The average accuracy was 2.3° of visual angle (s.e.: ± 0.1; range: 1.1−3.3). To assess inter-observer reliability for the categorization of fixations into areas of interest, a second coder, who was unaware of the study’s hypotheses, scored 20% of all trials (all categories: *Κ* = 0.86, *n* = 5427; only the bowls and face interest areas (IAs): *Κ* = 0.87, *n* = 1823).

To analyse the effectiveness of the directional cues, we analysed the maximal *x* coordinates in the baited direction in the signalling phase. We calculated this by determining the maximum or minimum *x* coordinate (depending on the baited side) in the signalling phase, subtracting the median *x* gaze coordinate of the addressing phase (baseline correction) and transforming the result into a proportion. For the fixation time analyses, we calculated the proportion time (sum of fixations divided by the duration of the interest period) the dogs looked into the different areas of interest.

We analysed the choice performance, i.e. whether the dogs chose the correct or incorrect cup (binary response variable) by fitting a binomial generalized linear mixed model (GLMM). We included as predictor variables condition (control, gazing, *p* + *g*, pointing, throwing), trial number within session (1−15), session number (1−2), age in months and sex. The covariates trial number, session number and age were *z*-transformed. We also included the random effect of subject ID as well as the random slopes of condition (centred), trial number and session number within-subject ID.

To analyse whether correct bowl looks in the signalling phase predicted correct subsequent choices, we extended the model by including the factor ‘correct bowl look’ (present/absent) and its interaction with the condition. We also included the random slope of the correct bowl looks within subject ID. Since the interaction was not significant, we refitted the model without the interaction to assess the main effects.

For the analysis of the proportions (looking time and maximal gaze coordinates in the baited direction), we fitted GLMM with beta error structure. We transformed the data so that they did not contain the extreme values 0 and 1 [38]. For the analysis of direct gaze transitions between the face, hand and correct bowl interest areas (IAs; gaze transitions coded as a binary variable, present/absent, within a trial) we fitted binomial GLMMs. We included the same predictor variables and random effects as in the choice model described above.

We drew inferences about the significance of fixed effect using the likelihood ratio test (R function *drop1* with argument test set to ‘Chisq’). Following a significant effect of condition, we conducted pairwise comparisons using Tukey contrasts to account for multiple testing (R package *multcomp*). Collinearity was no issue (max. variance inflation factor: <1.1). For the beta models, we also checked for overdispersion which was no issue either (dispersion parameter: GLMM 02: 1.01; GLMM03: 0.85; GLMM04: 0.76; GLMM05: 1.23). We checked the model stability by excluding each level of the random effect one at a time, refitting the model and comparing the resulting model estimates. This procedure revealed the models to be stable with respect to the fixed effects except for the ‘Face–Correct Bowl’ (GLMM07) and ‘Hand–Correct Bowl’ (GLMM09) transition models because in both cases, only a single dog showed such transitions in the control (GLMM07) and throwing (GLMM07 and 09) conditions.

Finally, we also compared dogs’ choice performance to a hypothetical chance value of 0.5. For this, we conducted a one-sample *t*‐test (two-tailed) on aggregated data using a Holm correction for multiple comparisons.

## 3. Results

### (a) Choice performance

**Figure 2.**
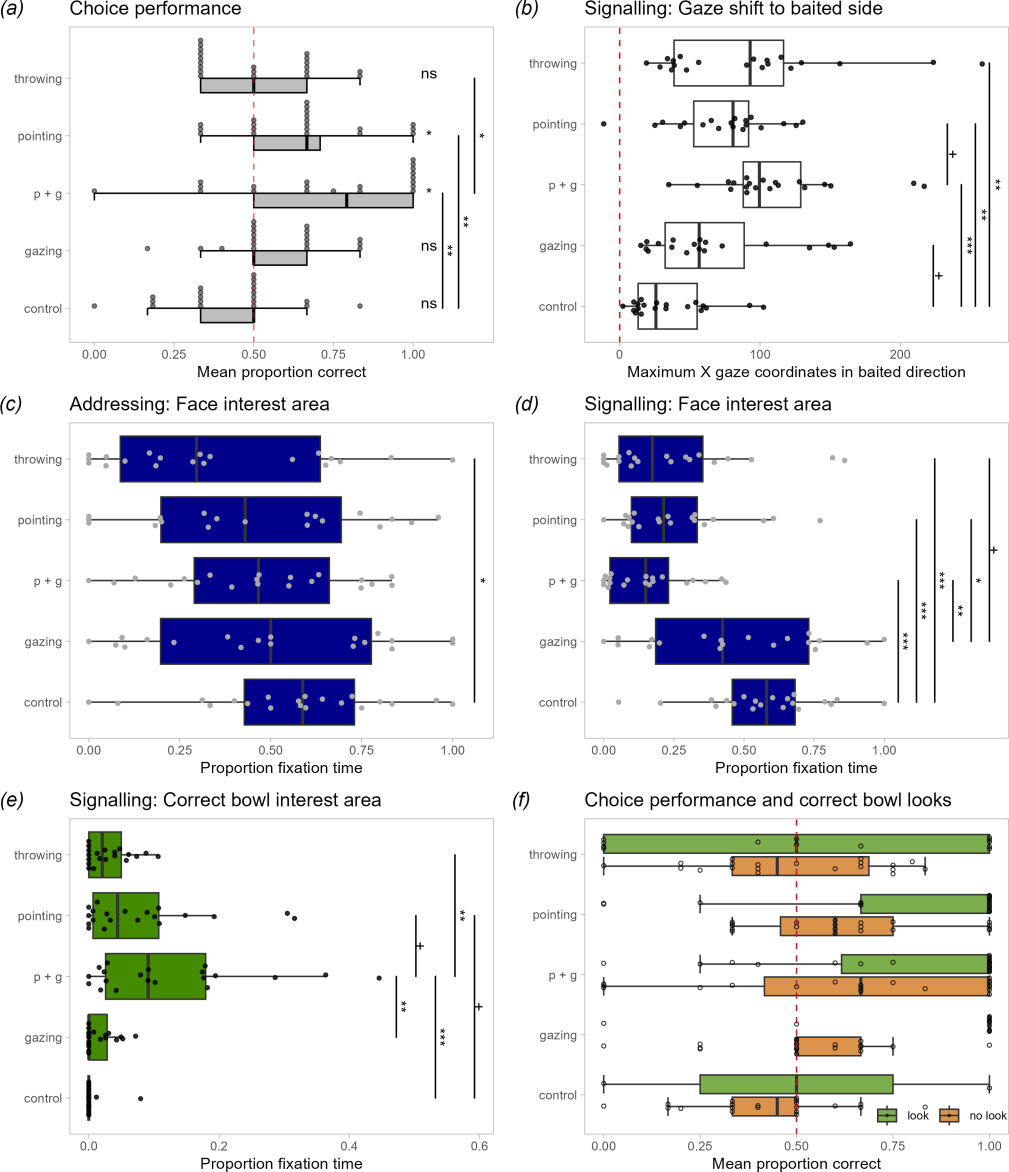
Box plots of the dogs’ choice and gaze behaviour across the five different conditions. The vertical lines inside the boxes represent the median, the boxes the interquartile range and the whiskers the range excluding outliers. The jittered dots represent the mean performance of the tested individuals. Asterisk and plus signs refer to the GLMM analysis (with the exception that in (*a*) some of the asterisks refer to one-sample *t*-tests, see below): +*p* < 0.1; *p < 0.05, **p < 0.01; ***p < 0.001; ns: non-significant (p > 0.1). (*a*) The dogs’ choice performance; plotted is the proportion correct, the dashed line represents the chance level and the asterisks close to the boxplots refer to one-sample *t*-tests comparing the dogs’ performance to the chance level. (*b*) The dogs’ maximal gaze shifts in the direction of the correct bowl in the signalling phase (corrected by their median horizontal gaze location in the preceding addressing phase). (*c*) The dogs’ proportion fixation time of the experimenter’s face interest area in the addressing phase and (*d*) in the signalling phase. (*e*) The proportion fixation time of the correct bowl in the signalling phase. (*f*) The dogs’ choice performance split across conditions and based on whether the dogs had looked at the correct bowl in the preceding signalling phase (shown in green) or not (orange). The dogs performed significantly better following correct bowl looks.

The dogs’ choice performance varied significantly between conditions (GLMM01: *χ*^2^(4) = 20.97, *p* < 0.001; mean ± s.e.: control: 0.42 ± 0.04; gazing: 0.57 ± 0.04; *p* + *g*: 0.72 ± 0.07; pointing: 0.64 ± 0.05; throwing: 0.50 ± 0.04). Pairwise comparisons (Tukey contrasts) revealed that the dogs performed significantly better in the *p* + *g* and pointing conditions than in the control condition and in *p* + *g* than the throwing condition (*p* < 0.05; see electronic supplementary material, tables S1, S2 and figure 2*a*); the other comparisons were not significant. The effects of trial number within a session, session number, age or sex were not significant (see electronic supplementary material, table S1). The dogs performed significantly better than expected by chance only in the *p* + *g* and pointing conditions (*p* < 0.05; Holm corrected; electronic supplementary material, table S3). This pattern is also reflected at the individual level: 8 out of 20 dogs performed significantly above chance in the *p* + *g* condition and 3 dogs in the pointing condition (6 out of 6 trials correct, binomial test: *p* < 0.05). None of the dogs performed significantly above chance in the other conditions.

### (b) Eye tracking

#### (i) Addressing phase

*Face IA*. The proportion fixation time to the face area did not vary significantly between conditions (GLMM02: *χ*^2^(4) = 8.52, *p* = 0.074; electronic supplementary material, table S4). Pairwise comparisons revealed that in the control condition, the dogs tended to look more at the experimenter’s face than in the throwing condition (*p* = 0.041), which can be attributed to the presence of the toy ball that the experimenter waved back and forth during the addressing phase. The other comparisons were not significant (electronic supplementary material, table S5). The other predictor variables, trial number, session number, age and sex, had no significant effect on the proportion fixation time to the face area (see electronic supplementary material, table S4).

#### (ii) Signalling phase

*Gaze movement in cued direction*. We first examined whether the dogs indeed followed the directional cues with their gaze. The maximal gaze movement in the cued direction during signalling varied significantly between conditions (GLMM03: *χ*^2^(4) = 38.65, *p* < 0.001; electronic supplementary material, table S6). All conditions with a hand cue (throwing, *p* + *g*, pointing) led to significantly more gaze shifts in the baited direction compared with the control condition (*p* < 0.05; electronic supplementary material, table S7). In the gazing condition, the dogs also tended to shift their gaze more in the baited direction than in the control condition (*p* = 0.052). The *p* + *g* tended to be more effective in shifting the dogs’ gaze than the pointing condition (*p* = 0.074). The dogs shifted their gaze more to the baited side in the second session than in the first (*χ*^2^(1) = 3.97, *p* < 0.05); the other control predictor variables trial number, age or sex had no significant effect on the gaze movement.

*Face IA*. The proportion fixation time to the face area varied significantly between conditions (GLMM04: *χ*^2^(4) = 62.27, *p* < 0.001; electronic supplementary material, table S8). Pairwise comparisons revealed that the dogs fixated on the experimenter’s face significantly longer in the control condition than in the conditions involving hand movements (*p* + *g*, pointing or throwing, *p* < 0.001; electronic supplementary material, table S9) but not compared with the gazing condition (*p* = 0.379). Likewise, in the gazing condition, the dogs looked significantly longer at the face area than in the pointing conditions (*p* + *g*, pointing, *p* < 0.05) and a similar trend was found for the comparison to the throwing condition (*p* = 0.063). The other comparisons were not significant. The other predictor variables, trial number, session number, age and sex, had no significant effect on the proportion fixation time to the face area (see electronic supplementary material, table S8).

*Correct bowl IA*. If the dogs looked at the correct bowl IA (or hand IA), they did so largely within the first second of the signalling phase (electronic supplementary material, figures S1 and S2). The proportion fixation time varied significantly among conditions (GLMM05: *χ*^2^(4) = 20.06, *p* < 0.001; electronic supplementary material, table S10). Pairwise comparisons revealed that the dogs fixated on the correct bowl significantly longer in the *p* + *g* condition than in the control, throwing and gazing conditions (*p* < 0.05; electronic supplementary material, table S11). The dogs also tended to fixate the correct bowl longer in *p* + *g* than in the pointing condition (*p* = 0.0998) and longer in the pointing than in the control condition (*p* = 0.084). The other predictor variables, trial number, session number, age and sex, had no significant effect on the proportion fixation time (see electronic supplementary material, table S10).

Next, we examined whether looks to correct bowls also predicted correct subsequent choices. We found no significant interaction between the condition and correct bowl looks (*χ*^2^(4) = 3.22, *p* = 0.521). When analysing the main effects, we found that the correct bowl looks significantly predicted correct subsequent choices (GLMM06: *χ*^2^(1) = 4.07, *p* = 0.044; electronic supplementary material, table S12). The dogs’ mean correct choice following correct bowl looks was 0.72 (s.e.: ± 0.05) compared with 0.54 (± 0.03) when they did not look at the correct bowl beforehand. As before (GLMM01), condition also had a significant effect on the choice performance (*χ*^2^(4) = 14.45, *p* = 0.006). The other predictor variables, trial number, session number, age and sex, had no significant effect on the proportion fixation time.

*Gaze transitions between areas of interest*. Visualizing the time-series data with respect to the fixated areas of interest in the signalling phase suggests that there are clear differences between the conditions that are not limited to the comparison between the cue conditions and the no-cue control (see electronic supplementary material, figure S1).

**Figure 3.**
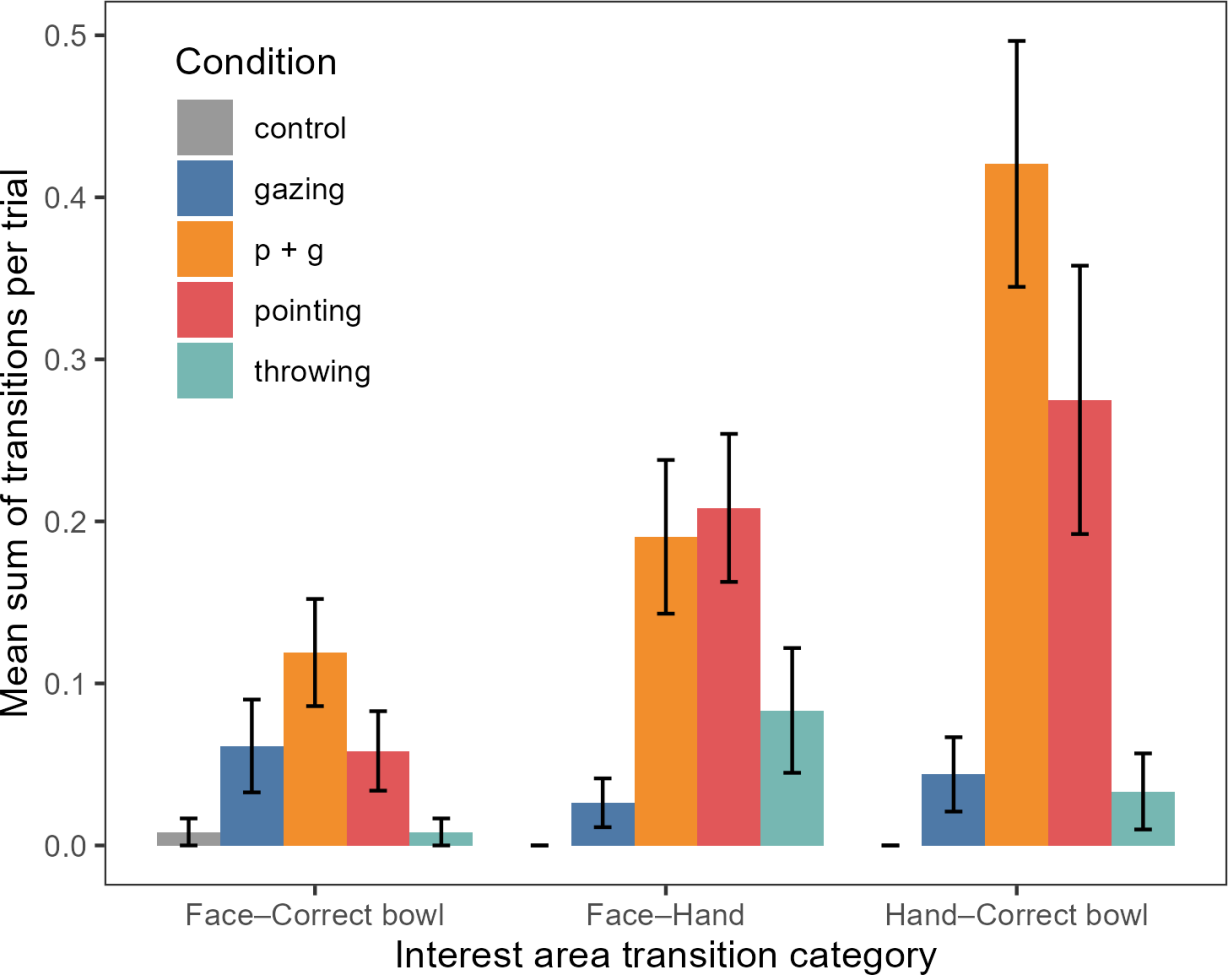
Bar plot showing the mean sum of gaze transitions per trial (signalling interest period) across conditions. The gaze transitions between areas of interest include ‘Face–Hand’, ‘Face–Correct Bowl’ and ‘Hand–Correct Bowl’. The error bars refer to the s.e. of the mean.

The frequency of gaze transitions between the face IA and correct bowl IA varied significantly between conditions (GLMM07: *χ*^2^(4) = 14.76, *p* = 0.005; electronic supplementary material, tables S13 and S14; figure 3). However, pairwise comparisons accounting for multiple comparisons did not reveal any of the comparisons to be significant even though the dogs tended to shift their gaze more often between face and correct bowl in the *p* + *g* condition than in the control (*p* = 0.078) or throwing condition (*p* = 0.075).

The frequency of gaze transitions between the face IA and hand IA varied significantly between conditions (we did not include the control condition here due to complete separation issues, i.e. no face–hand transitions in the control condition; GLMM08: *χ*^2^(3) = 10.80, *p* = 0.013; electronic supplementary material, tables S15 and S16; figure 3). The dogs shifted their gaze significantly more often in between face and hand in the *p* + *g* (*p* < 0.050) and pointing condition (*p* = 0.029) than in the gazing condition.

Finally, the frequency of gaze transitions between the hand IA and correct bowl IA also varied significantly between conditions (again the control condition data were excluded here due to complete separation issues, i.e. no hand–correct bowl transitions in the control condition; GLMM09: *χ*^2^(3) = 23.57, *p* < 0.001; electronic supplementary material, tables S17 and S18; figure 3). The dogs shifted their gaze significantly more often in between hand and correct bowl in the *p* + *g* condition than in the gazing (*p* < 0.001) or throwing condition (*p* = 0.015) and by trend also when compared with the pointing condition (*p* = 0.068).

## 4. Discussion

Our results show a clear difference in how dogs reacted to the directional but non-referential throwing cue compared with the referential hand gestures. While all cues including hand movements reliably prompted dogs to look to the side (unlike the referential gaze cue), only when presented with a pointing gesture did dogs redirect their gaze from the experimenter’s hand to the designated bowl. The combination of gazing and pointing cues was particularly effective in directing the dogs’ attention to the baited bowl and influencing their subsequent choices. In the pointing + gazing condition, the dogs shifted their gaze most frequently from the experimenter’s face to her hand and from the hand to the correct bowl (figure 3 and electronic supplementary material, figure S1). When the dogs looked at the baited bowl, they were in general more likely to select this bowl, in line with previous research [34]. However, the dogs’ gaze shifts merely towards the *side* of the baited bowl alone were not sufficient to bias their subsequent choices: in the fake throwing condition, the dogs’ gaze was moved towards the baited side to a similar extent as in the pointing conditions but not to the precise location of the correct bowl and it did not lead them to choose the correct bowl significantly above chance levels. In general, the gaze shifts between the head of the communicator, the pointing hand and the referent in the pointing conditions suggest that dogs are sensitive to the referentiality of these communicative hand gestures.

In a previous screen-based eye-tracking study [22], the contrast between ostensive and non-ostensive cues was in focus. In the current study, we focused on different signals within the context of ostensive communication. We found that gaze cues, even if administered in a repeated fashion and interpreted as referential communication by humans, did not have a clear effect on dogs’ attention towards and subsequent choice of the signalled container. We note that in the previous (screen-based eye tracking) study [22], the dogs were trained to look at the containers in the warm-up phase of the experiment, which might have increased their tendency to follow the gaze cues in the test phase. Our results are in line with other studies, using no such pretraining, that also found that dogs follow gaze cues to a lesser extent than pointing. The authors of these studies have suggested that dogs interpret directional gaze as an intentional cue rather than a communicative signal [16,17]. We extend their findings by showing how the combination of pointing and gazing (in contrast to the individual cues) is particularly effective in directing the dogs’ gaze to a referent. Our controls exclude other cues (e.g. olfactory cues) and also that any directional action would be insufficient to shift the dogs’ gaze to the referent.

It is possible that the two different communicative signals (gazing and pointing) simply added up and thereby increased the likelihood of shifting the dogs’ attention, but it is also possible that the two cues work synergistically, exceeding the sum of their individual effects on dogs’ attention and subsequent choice behaviour. For example, alternating gaze between a referent and the dogs, especially if accompanied by pointing, may serve as a maintained ostensive signal. These findings may also explain why previous studies have struggled to find robust evidence for gaze and point following when these cues were presented in isolation [33]. The combination of these cues seems key.

Apart from such additive or synergistic effects, dogs might find it harder to disengage from eye contact with the communicator when the communicator is looking constantly at the dog as in the pointing condition [39]. The gazing cue in the *p* + *g* condition might have facilitated the dogs’ gaze shift to the hand and from there to the correct bowl, not directly due to the gazing cue but due to interrupted eye contact with the dog. Indeed, the dogs’ fixation sequence suggests that dogs often shifted their gaze in this sequence (electronic supplementary material, figure S1).

Eye-tracking studies with humans also revealed additive effects of pointing and gaze cues: children with autism spectrum disorder were found to pay little attention to the referent of pointing gestures [40] and they also show reduced levels of gaze following [41]. However, presented with combined pointing and gaze cues, children with autism spectrum disorder were significantly more likely to look at the referent [42].

In contrast to screen-based studies, mobile eye tracking provided the advantage of monitoring dogs’ gaze behaviour in a more ecologically valid, real-world scenario involving real social interactions. Thereby, we could capture their spontaneous reactions to human cues, reflecting how they would respond in everyday interactions with humans. We could record their gaze shifts basically in real time, showing precisely how and when their attention shifted from the experimenter’s hand or face to the correct bowl. The temporal patterns of gaze shifts revealed not only whether dogs ultimately made a correct choice but also how they took up the information leading up to that choice. For instance, in *p* + *g*, dogs more quickly disengaged their attention from the human’s face and looked more quickly to the hand and the correct bowl than in the other conditions (electronic supplementary material, figures S1 and S2). Examining the timing of the gaze shifts, especially in the initial seconds of the signalling phase, provided evidence that early looks were critical for their subsequent decision-making supporting the notion that real-time monitoring of gaze patterns can inform us about how dogs prioritize and filter information.

In summary, mobile eye tracking provided evidence that after ostensive addressing dogs selectively follow certain directional cues to the indicated referent. The combination of pointing and gazing cues proved most effective in manipulating their overt visual attention as well as their subsequent choice behaviour. Pointing without gaze cues also affected the dogs’ attention and choice performance even though to a lesser extent whereas gaze cues alone were ineffective. Fake throwing had little effect on the dogs’ attention or choice behaviour either and in contrast to the conditions involving pointing and or gazing, even if the dogs looked at the target bowl following the fake throwing this did not lead them to preferentially choose this bowl. This study confirms the finding that dogs follow a referential cue to a target more than a directional cue alone [21]. Finally, the current study showed that mobile eye tracking can provide valuable insights into how dogs navigate their environment including the interactions they have with their human partners.

According to the ‘natural pedagogy’ framework [43], which aims to explain how humans acquire cultural knowledge, ostensive signals in humans do not only increase attention but also elicit a referential expectation and a genericity bias. Eye tracking could be used in the future to study whether ostensive cueing of an object also affects how dogs process information about this object, that is, whether ostensive signalling leads to better or qualitatively different memory encoding of the signalled object. Identity change paradigms have been used with human infants to study this issue in preverbal participants [44–46]. Following this approach, future studies might focus on whether jointly attending objects or locations not only affects dogs’ gaze behaviour but also whether it affects their subsequent information processing and memory encoding.

Mobile eye tracking could also be used in other dog populations, including puppies and shelter dogs, to shed more light on the ontogeny of their understanding of human referential communication. We know that dogs with varying levels of exposure to humans perform worse than pet dogs in studies of pointing comprehension [4–9]. It would be interesting to examine whether such differences are also found in their gaze following in response to human referential communication or whether their attention is similar even though it does not translate into choice performance. It would also be interesting to see in longitudinal studies either involving training or experience with humans [47] whether correct looks precede correct choices or whether they coincide over the course of the training. Finally, mobile eye tracking will provide information on how dogs with specific training backgrounds (such as guide dogs for the blind) respond to human communication and, more generally, how their attention differs from dogs without such specific training backgrounds.

## Data Availability

All data and code used in the analyses are available on a public repository [48]. Supplementary material is available online [49].
